# Autoimmune Aspects of Neurodegenerative and Psychiatric Diseases: A Template for Innovative Therapy

**DOI:** 10.3389/fpsyt.2017.00046

**Published:** 2017-04-04

**Authors:** Peter de Haan, Hans C. Klein, Bert A. ’t Hart

**Affiliations:** ^1^Amarna Therapeutics B.V., Leiden, Netherlands; ^2^Department of Psychiatry and Medical Imaging Centre, University Medical Centre Groningen, Groningen, Netherlands; ^3^Department of Immunobiology, Biomedical Primate Research Centre, Rijswijk, Netherlands

**Keywords:** neurodegenerative disease, psychiatric disease, chronic inflammation, immune tolerance, self-antigen, viral vector, reverse vaccine

## Abstract

Neurodegenerative and psychiatric diseases (NPDs) are today’s most important group of diseases, surpassing both atherosclerotic cardiovascular disease and cancer in morbidity incidence. Although NPDs have a dramatic impact on our society because of their high incidence, mortality, and severe debilitating character, remarkably few effective interventions have become available. The current treatments, if available, comprise the lifelong intake of general immunosuppressants to delay disease progression or neurotransmitter antagonists/agonists to dampen undesired behaviors. The long-term usage of such medication, however, coincides with often severe adverse side effects. There is, therefore, an urgent need for safe and effective treatments for these diseases. Here, we discuss that many NPDs coincide with subtle chronic or flaring brain inflammation sometimes escalating with infiltrations of lymphocytes in the inflamed brain parts causing mild to severe or even lethal brain damage. Thus, NPDs show all features of autoimmune diseases. In this review, we postulate that NPDs resemble autoimmune-driven inflammatory diseases in many aspects and may belong to the same disease spectrum. Just like in autoimmune diseases, NPD symptoms basically are manifestations of a chronic self-sustaining inflammatory process with detrimental consequences for the patient. Specific inhibition of the destructive immune responses in the brain, leaving the patient’s immune system intact, would be the ultimate solution to cure patients from the disease. To reach this goal, the primary targets, e.g., the primary self-antigens (pSAgs) of the patient’s chronic (auto)immune response, need to be identified. For a few major NPDs, immunological studies led to the identification of the pSAgs involved in the autoimmune damage of specific brain parts. However, further research is needed to complete the list of pSAgs for all NPDs. Such immunological studies will not only provide crucial insights into NPD pathogenesis but also ultimately enable the development of a new generation of safe and effective immunotherapies for NPDs. Interventions that will dramatically improve the life expectancy and quality of life of individual patients and, moreover, will significantly reduce the health-care costs of the society in general.

## The Cause of Degenerative Diseases

The immune system comprises an intricate network of tissues, cells, and molecules responsible for responding to pathogen infection, malignant cells, and wounding, and thus for maintaining the body’s homeostasis.

The first line of active immunity, named innate immunity, is activated by general evolutionary conserved pathogen-associated molecular patterns (PAMPs) released from infected cells or damage-associated molecular patterns (DAMPs) released by malignant and wounded cells (1). Cells of the innate immune system carry a variety of cytoplasmic and membrane-bound pattern recognition receptors to sense and respond to PAMPs and DAMPs. Upon binding of these alarm signals, the innate immune cells remove the cognate pathogens, damaged, or malignant cells and, when necessary, activate the second line of immunity, named adaptive immunity. Our adaptive immune system consists of humoral and cellular components. Humoral adaptive immunity is mediated by B lymphocytes (B cells), which secrete antibodies, whereas cellular adaptive immunity is mediated by T lymphocytes (T cells), which secrete cytokines and are capable of destroying damaged, malignant, or infected cells. The adaptive immune system acts *via* the activation of antigen-specific cell clones, which means that each clone of B or T cells bears a different receptor that binds antigenic peptides (epitopes) with a high specificity. Where B cells directly bind antigen epitopes, recognition by T cells requires presentation of antigen epitopes on major histocompatibility complex molecules.

During homeostasis, the adaptive immune system is in the tolerance mode. Immune tolerance is actively maintained by homeostatic interactions between somatic cells and innate immune cells with lymphocytes wherein the lymphocytes have a regulatory role by suppressing immune responses to self and foreign antigens (2, 3).

When PAMPs or DAMPs released by infected, wounded, and malignant cells are perceived by innate immune cells, inflammasomes assemble in the cytoplasm and a local inflammatory response is initiated. Inflammation is a highly orchestrated cascade of protective local and systemic events aimed at confining the pathogen, reducing the cell damage, promoting wound repair, and removing malignant cells (4). When all necroptotic cellular components, PAMPs, and DAMPs are removed, the inflammation stops and the homeostasis is restored. Usually, in healthy individuals, the repair activity of the innate immune system is sufficient to maintain homeostasis.

However, in the case the infection is too widespread, the wound is too big or the malignancy grows too fast, and the innate immune cells are not able to remove all the necroptotic cellular components, PAMPs, and DAMPs in time, cells of the adaptive immune system will infiltrate the inflamed area, locally break the immune tolerance in an antigen-specific manner, and destroy the infected, injured, or malignant cells (5). The destroyed and thus necroptotic cells further enhance the cellular immune response and speed-up the wound repair process (6). Again, after such a normal wound repair response, when all necroptotic cellular components, PAMPs, or DAMPs are removed, inflammation stops and the tolerance toward self will be restored (7).

In the case an adaptive immune response to self for whatever reason remains active, the wound repair process becomes self-sustaining and chronic. Chronic or flaring immune responses to tissue-specific antigens coincide with amyloid plaque formation, hypervascularization, fibrosis, and tissue scarification (jointly named sclerosis) at the site of inflammation. Wound healing is a beneficial process, but chronic self-sustaining wound healing is detrimental to the affected tissue and will result in the development of a degenerative disease (8–11). In this process, the self-antigen-specific chronic or repeated stimulation of a cellular immune response is the cause and driver of pathogenesis and thus disease progression (12).

The incidence of degenerative diseases associated with chronic or flaring immune responses, such as obesity, diabetes mellitus, arthritis, atherosclerotic cardiovascular disease, and NPDs, is increasing rapidly in our aging population. The reason for this increase has remained unclear. With a global population of more than seven billion individuals, we are a highly successful mammalian species. Several hypotheses have been formulated to explain our success. One of them says that we have developed an extremely efficient immune system that enables us to reach a high age under extremely challenging biotic and abiotic environmental conditions. The down-side of having such an efficient or “tensed” immune system is the increased risk of developing undesired immune responses to self-components, resulting in autoimmune diseases, or to harmless non-self-components, resulting in allergies.

## The Cause of the Cause of Degenerative Diseases

The majority of degenerative diseases are sporadic (idiopathic or acquired) diseases, and because the immune responses in patients are directed to tissue-specific self-antigens, these diseases are classified as autoimmune diseases. Examples of well-studied autoimmune diseases include diabetes mellitus type 1 (DM1), rheumatoid arthritis (RA), and multiple sclerosis (MS). For these diseases, the causal relationships between chronic immune activation, pathogenesis, and disease symptom development have been demonstrated in experimentally induced animal models. Autoimmune diseases are the result of an interplay between environmental stimuli, such as diet, lifestyle, trauma, exposure to microbes, pathogens, or toxic compounds (smoking), and the possession of predisposing gene alleles, which ultimately at a certain moment in time “trigger” a sustained loss of self-tolerance resulting in an immune-mediated damage of autologous tissues (13). Frequently, patients with an autoimmune disease have an increased susceptibility to develop other autoimmune diseases. Genes involved in innate or adaptive immunity, such as antigen uptake, processing, presentation, and signaling, represent the strongest predisposing genetic factors (14). This indicates that exposure to environmental trigger stimuli is the cause of aberrant immune responses to self-antigens, which on its turn is the cause of the development of the characteristic disease symptoms.

In patients with a pathogen-induced degenerative disease, such as viral hepatitis and the acquired immune deficiency syndrome, the causal trigger stimuli are chronic infections resulting in a continuous release of PAMPs by the infected and necroptotic cells. This results in chronic self-sustaining immune reactions directed against antigens derived from the pathogen. In case the pathogen-derived antigens mimic self-antigens (molecular mimicry), a chronic immune response will be induced in the tissue that expresses the mimicked self-antigens. There are indications that retroviral elements and chronic infections with herpesviruses such as Epstein–Barr virus or cytomegalovirus are associated with a number of autoimmune diseases, including NPDs with relapsing and remitting phases. Harmless bacteria may elicit an erroneous chronic immune response. In principle, such a response to harmless microbe-derived antigens is an allergic response. When the microbe-derived antigens mimic self-antigens, autoimmune diseases may be induced in predisposed individuals. Examples of bacteria-derived NPDs are the Guillain–Barré syndrome caused by *Campylobacter jejuni* and Lyme disease caused by tick-borne *Borrelia burgdorferi* infections.

The majority of degenerative diseases, however, result from an improper restoration of immune tolerance to specific self-antigens after a normal wound repair response. The incapacity to restore immune tolerance in a particular organ or tissue may occur when DAMPs are over-abundantly released inducing a hyperimmune response, when DAMPs continuously keep emerging, or when lymphocytes and tissue cells interact aberrantly during a wound repair response.

In humans with a degenerative disease, it is almost impossible to identify the primary targets of the activated T cells (the primary self-antigens, pSAgs), since the sustained destructive immune responses manifest often years after the onset, basically as the characteristic disease symptoms resulting from the tissue damage. Most efforts to identify the pSAgs of a degenerative disease have been dedicated to verifying humoral immune responses in patients with the disease. These studies have revealed the presence of autoantibodies in the sera of patients that usually bind to intracellular components which are released in the blood after tissue degeneration. Because the studies are performed with blood samples from patients who have the disease for a relatively long time, many of the identified antibody targets represent secondary self-antigens, which may also be post-translationally modified by the pathogenic process.

Acute autoimmune encephalitis is a rare group of NPDs diagnosed by the presence of autoantibodies in the sera of patients that bind hippocampal self-antigens (15). In approximately half of the cases, the encephalitis is caused by the presence of tumors in patients that express and present neo-antigens that are identical to the hippocampal self-antigens. In the other half of the cases, it is assumed that infections with unknown viruses or microbes are the cause of the encephalitis (16, 17).

For a long time, the role of cellular immunity in the tissue destruction has been underestimated. Only recently, it has become clear that T cells play a predominant role in the tissue destruction in patients with a degenerative disease. For a number of degenerative diseases, studies on the variability of T cells that infiltrate the inflamed tissue of a patient, like, for example, the anterior horns of the spinal cords of amyotrophic lateral sclerosis (ALS) patients and the hippocampus of epilepsy patients, revealed that the infiltrating T cells comprise a single or few cell clones, indicating that a cellular adaptive immune response to one or few self-antigens is responsible for the autoimmune tissue damage (18, 19). Therefore, immunological studies focusing on cellular immune responses to self-antigens, preferably in early onset patients, may be the preferred method to identify the pSAgs of the disease.

A more feasible approach to demonstrate the causal relationship between chronic immunity to a pSAg and disease symptom development is to immunize animals with a pSAg of a degenerative disease. Immunization of animals with a secondary self-antigen does not lead to the induction of disease symptoms, because there is a massive immune tolerance present and actively maintained to these proteins in the non-inflamed body parts. The now classical immunization experiments with pSAgs have not only led to the identification of the pSAgs of degenerative diseases but also to the development of the currently used and most valuable inducible animal models of human degenerative diseases, such as the myelin oligodendrocyte glycoprotein (MOG)/myelin basic protein (MBP)-induced rodent and marmoset experimental autoimmune encephalitis (EAE) models resembling human MS, the proinsulin-induced mouse model of human DM1 and the collagen type 2-induced rodent and marmoset collagen type 2-induced arthritis models resembling human RA. The pSAgs of degenerative diseases identified thus far are tissue-specific extracellular matrix proteins, cytoskeleton-associated proteins, or components involved in intercellular signaling. Secondary self-antigens are generally cytoplasmic proteins present in different tissues and are targeted relatively late by the patients’ immune system when the affected tissue is chronically inflamed in a process referred to as epitope spreading.

In analogy to the Koch’s postulates for identifying the causal relation between pathogens and infectious diseases, pSAgs of degenerative diseases have to fulfill the following three criteria:

1. the pSAg of a degenerative disease is predominantly or exclusively expressed in the affected tissue.
2. induction of an adaptive immune response to the pSAg in an animal results in the development of the disease.
3. all patients have an adaptive immune response to the pSAg, while these are absent in healthy people.

With the exception of MS, the role of the immune system in NPD pathogenesis is relatively poorly studied. Only for a few NPDs solid immunological studies in animals and patients have been employed to identify the pSAgs of the disease. Immunological follow-up studies for other NPDs are highly desired, because they will not only provide key insights into the pathogenesis of this group of diseases but also result in new targets for drug development.

## The Candidate pSAgs of NPDs

Neurodegenerative and psychiatric diseases are associated with chronic or flaring inflammation of specific brain areas with infiltration of peripheral immune cells, resulting in mild or severe brain damage that leads to the development of the characteristic disease symptoms. Based on the type of neurotransmitter used for signaling by the affected neurons the diseases can be categorized in groups (See Table 1).

**Table 1:** **Candidate primary self-antigens (pSAgs) of neurodegenerative and psychiatric diseases**.

Group	Neuron type	Disease	Affected brain part	pSAg
1	Cholinergic	Multiple sclerosis	White matter	Myelin oligodendrocyte glycoprotein, myelin basic protein
		Alzheimer’s dementia	Basal forebrain	Neurofilament heavy
		Amyotrophic lateral sclerosis	Motor neurons	Neurofilament light
		Autism spectrum disorder (autism, Asperger syndrome, PDD-NOS)	Striatum, cerebellum	Muscarine acetyl choline receptors
2	Dopaminergic	Huntington disease	Striatum	Huntingtin
		Parkinson disease	Substantia nigra compactum and VTegm	Vesicular monoamine transporter-2
		Attention-deficit hyperactivity disorder	Mesostriatal system	Dopamine transporter-1
		Reward deficiency syndrome (addiction, obsessive–compulsive disorder)	Striatum	Dopamine receptor-1, 2, 3
3	Glutamatergic	Epilepsy (temporal lobe epilepsy)	Hippocampus	metabotropic Glutamate receptor 3
		Schizophrenia	Hippocampus	N-methyl-D-aspartate receptor
4	Histaminergic	Tic diseases (Tourette syndrome)	Basal ganglia	Histamine 3 receptor
5	Serotonergic	Depression	Prefrontal cortex	5-hydroxytryptamine transporter
		Anxiety	Brain stem	5-hydroxytryptamine receptor 2C

The identification of the candidate pSAgs is based on the combination of different lines of research as indicated in Section “The Candidate Primary Self-Antigens of NPDs.”

In patients with a familial neurodegenerative disease, such as Huntington disease (HD), and the familial forms of Alzheimer’s dementia (AD), Parkinson disease (PD), ALS, and epilepsy with accumulated genetically predestinated cellular defects, DAMPs originating from necroptotic cells continuously emerge in a specific brain part. As a consequence of this, a self-sustaining immune response to tissue-specific self-antigens is induced in the brains of patients. However, a major part of the neurodegenerative diseases (and all psychiatric diseases) is sporadic, meaning that the trigger environmental stimulus is unknown.

Neurodegenerative diseases are the result of the presence of predisposing gene alleles (risk genes) interacting with unknown environmental stimuli, such as trauma, infection, or exposure, to toxic compounds. The immune reactions in neurodegenerative diseases (HD, PD, MS, AD, ALS, and epilepsy) usually are severe and associated with massive amyloid deposition (huntingtin, alpha-synuclein, Abeta, tau, TDP-43, etc.) and sclerosis at the affected brain part, often resulting in a fatal loss of function.

Psychiatric diseases are the result of the presence of risk genes determining the personality interacting with environmental stimuli, such as psychosocial factors, lifestyle, and stress. In contrast to neurodegenerative diseases, the CNS inflammation and immunity in the psychiatric diseases is mild, with barely detectable damage to the affected brain part [attention-deficit hyperactivity disorder (ADHD), addiction, reward deficiency syndrome (RDS), depression, anxiety, autism spectrum disorder (ASD), schizophrenia, and Tic disease (TD)] (20).

Neurodegenerative and psychiatric diseases can be progressive (HD, PD, ADHD, RDS, AD, ALS, and ASD) or relapsing-remitting (anxiety, depression, MS, epilepsy, schizophrenia, and TD). For these diseases, the immune responses flare. During relapses, the immune tolerance is broken and the symptoms are worse and during remissions, the immune tolerance is intact and the disease does not progress.

For the majority of NPDs, the pSAgs involved in the brain damage await identification and there is only indirect evidence what they could be (See Table 1, last column). For this latter group of diseases imaging, pharmacological and genetic studies using knockout and knockdown mouse mutants revealed a number of candidate pSAgs, but immunological studies as outlined in the previous paragraph are needed to confirm their role in the disease pathology.

### Group 1: Cholinergic NPDs

#### Multiple Sclerosis

Multiple sclerosis is characterized by chronic inflammation and degeneration of cholinergic myelinated axons in the brain and spinal cord, resulting in functional disability and premature death (21). Analysis of the T cell receptors (TCRs) present on the surface of cytotoxic T lymphocytes (CTLs) that infiltrate the inflamed brain parts revealed that these CTLs have a strongly biased TCR repertoire compared to splenic CTLs. This suggests that the infiltrating CTLs recognize a single or few self-antigens (8, 22). Immunization of animals with MOG or MBP or peptides derived thereof results in the development of EAE that shares similarities with human MS (23). Since MOG and MBP fulfill the three criteria outlined in Section “The cause of the cause of degenerative diseases,” these two proteins are candidate pSAgs involved in the autoimmune inflammatory/demyelination of cholinergic neurons in the CNS white matter MS patients.

#### Alzheimer’s Dementia

Alzheimer’s dementia is the most common cause of aging-related dementia, associated with chronic inflammation and T lymphocyte-mediated degeneration of cholinergic myelinated large axons in the basal forebrain, resulting in severe cognitive impairment and eventually death (24–26). Rats immunized with adjuvated extracts of cholinergic neurons from the electric fish, Torpedo, develop experimental autoimmune dementia (EAD) resembling human AD (27). It was found that the intermediate neurofilament heavy (NFH) protein is the most likely pSAg in these extracts, capable of inducing EAD in rats (28). Almost all diagnosed AD patients have a humoral immune response to NFH, but not to the intermediate neurofilament light (NFL) protein (29, 30). Since NFH fulfills the three criteria outlined in Section “The cause of the cause of degenerative diseases,” this neurofilament is a candidate pSAg involved in the autoimmune cortex destruction in AD patients.

#### Amyotrophic Lateral Sclerosis

Amyotrophic lateral sclerosis is characterized by progressive muscle weakness (paresis), disability, and eventually death, with a median survival of 3–5 years. The motor cortex and the anterior horn of the spinal cord of ALS patients are chronically inflamed and infiltrated with macrophages and CTLs, resulting in the loss of cholinergic myelinated motor axons (31–35). Immunization of guinea pigs with adjuvated bovine motor neuron extracts induces experimental autoimmune motor neuron disease (EAMD) resembling human ALS (36). Immunization of mice with NFL results in the development of spastic paresis resembling EAMD (37). It has further been reported that almost all diagnosed ALS patients have antibodies against NFL (38–40). Since NFL fulfills the three criteria outlined in Section “The cause of the cause of degenerative diseases,” it is believed that neurofilament is a candidate pSAg involved in the autoimmune destruction of motor neurons in ALS patients.

#### Autism Spectrum Disorders

Autism spectrum disorder, including autism, the Asperger syndrome and the pervasive developmental disorder-not otherwise specified (PDD-NOS) represent a group of NPDs involving cholinergic neurons in the dorsomedial striatum and cerebellum characterized by often severe repetitive behaviors and cognitive inflexibility manifested as an impaired development of social interaction and communication skills and a markedly restricted repertoire of activity and interest. ASD coincides with chronic inflammation and altered immune responses in the affected brain parts (41–43). Studies in rats demonstrated that antagonists of muscarine acetyl choline receptors (mAChRs) increase and mAChR agonists reduce the repetitive behaviors (44, 45). Treatment of mouse models of human ASD with acetylcholine esterase inhibitors resulting in increased synaptic acetylcholine levels leads to an improvement of the behavioral rigidity (46). Destruction of cholinergic neurons in the dorsomedial striatum leads to increased repetitive behaviors that can be alleviated by treatment with M1 mAChR agonists (47), suggesting that the loss of cholinergic signaling by the autoimmune damage to the M1 mAChR-bearing neurons in the dorsomedial striatum results in the development of ASD.

### Group 2: Dopaminergic NPDs

#### Huntington Disease

Huntington disease is a progressive and usually fatal neurodegenerative disease of the striatum associated with the gradual loss of dopaminergic striatal neurons involved in motor coordination and subcortical dementia. The disease is caused by mutations in the gene encoding huntingtin, resulting in the chronic deposition of huntingtin amyloid in the striatum that coincides with a chronic immune response to a striatum-specific self-antigen (possibly huntingtin) and a gradual loss of dopaminergic striatal neurons (48).

#### Parkinson Disease

Parkinson disease is the most common neurodegenerative disease at younger age. PD symptoms include tremor, uncontrolled movements (bradykinesia), muscle stiffness, impaired posture and balance, loss of automatic movements, addiction, and loss of cognitive functions. PD coincides with chronic inflammation and T lymphocyte infiltration of the dopamine-producing neurons in the substantia nigra compactum and ventral tegmentum (49) and a gradual loss of dopamine-producing neurons (50–52). Immunization of mice or guinea pigs with dopaminergic neuronal cells or substantia nigra neurons results in the development of experimental autoimmune nigral damage with symptoms resembling human PD (53, 54). PD has been associated with the loss of vesicular monoamine transporter-2 (VMAT2) in dopamine-producing neurons resulting in elevated levels of cytoplasmic dopamine, which is highly neurotoxic (55, 56). Injection of animals with the VMAT2 antagonist 1-methyl-4-phenyl-1,2,3,6-tetrahydropyridine (MPTP) leads to necroptosis of dopamine-producing neurons due to the accumulation of neurotoxic dopamine in the cytoplasm. The necroptotic dopamine-producing neurons subsequently induce a chronic T cell-mediated and nigral-specific immune response that results in the development of the characteristic PD symptoms (51, 57). MPTP-induced PD in mice and marmosets are generally considered the most accurate animal models of human PD. VMAT2-knockout mice display the pathology and symptoms of PD, whereas VMAT2-knockdown mice are highly susceptible to MPTP-induced PD induction (58). On the contrary, animals overexpressing VMAT2 have increased striatal dopamine levels and are MPTP-resistant (59). These genetic, pharmacological, and immunological studies strongly suggest that VMAT2 expressed at high levels in dopamine-producing neurons is a candidate pSAg involved in the autoimmune destruction of dopamine-producing neurons in PD patients (60).

#### Attention Deficit Hyperactivity Disorder

Attention-deficit hyperactivity disorder symptoms include difficulties staying focused and paying attention, impulsivity (difficulty in controlling behavior), and hyperactivity. ADHD patients have inflammation markers in the blood (61) and white matter loss in the striatal, frontal, and parietal, dopamine-innervated brain areas (62–64). Mice with knockout and knockdown mutations in the dopamine transporter-1 (DAT1) gene show severe behavioral changes, including hyperactivity, memory, repetitive behavior, and learning deficits, mimicking human ADHD (65–68). Immunization of mice with DAT1 results in the development of characteristic ADHD symptoms (64). In addition, ADHD patients have a humoral immune response to DAT1 (69). Since DAT1 fulfills the three criteria outlined in Section “The Candidate Primary Self-Antigens of NPDs,” it is believed that transporter is the pSAgs involved in the autoimmune damage of the dopaminergic striatal and cortical neurons in ADHD patients. Furthermore, these studies suggest that DAT1 is predominantly expressed in post-synaptic neurons in the striatum and frontal–parietal brain areas that are innervated by dopamine-producing neurons.

#### The Reward Deficiency Syndrome

Reward deficiency syndrome comprises substance addiction and the obsessive–compulsive disorder (OCD). Substance addiction resides in the dopaminergic neurons of the nucleus accumbens in the striatum and coincides with increased inflammation markers in the affected compartment (70, 71). The chronic and disabling obsessional thoughts and compulsive rituals of OCD patients are associated with hyperactivity of the ventral cognitive circuit, involving dopaminergic neurons in the striatum (72, 73). OCD coincides with inflammation markers in the blood and the striatum (74–77). Drug use leads to elevated levels of dopamine, increased dopamine receptor-1 (DAR1), and decreased DAR2 signaling in the striatum that results in stimulation of the reward circuitry (78). In drug addicts, striatal DAR2 levels are reduced compared to those in healthy individuals (79). Mutations in the gene encoding DAR2 which render the receptor less sensitive to dopamine are associated with addictive behavior (80). Downregulation of DAR2 in rats promotes the reward deficits resulting in addictive behavior, such as compulsive food-seeking (81). DAR2 knockout mice display elevated DA synthesis resulting in hyperlocomotion and supersensitivity to drugs, such as cocaine (82). DAR2 overexpression in the nucleus accumbens of rats attenuates addictive behavior (83). These studies together with genetic and pharmacological studies on DAR3 (84–86) suggest that a dopamine receptor from the DAR2 family is the most likely candidate pSAg of addiction. Pharmacological studies in mice using dopamine receptor-1 agonists show that these drugs induce complex movement sequences such as grooming, resembling human OCD (87). Overall, the studies on dopamine receptors suggest that they are the candidate pSAgs for the reward-associated NPDs, addiction, and OCD.

### Group 3: Glutamatergic NPDs

#### Epilepsy

Epilepsy is a group of diseases characterized by convulsive or absence seizures, caused by an improper functioning of glutamatergic cortical neuron ion channels. The most common form of epilepsy is temporal lobe epilepsy (TLE) in which the seizures occur in the hippocampus of patients. Repeated seizures in epilepsy patients are associated with immune responses, resulting in hippocampal necroptosis and/or cortical damage at the site of the seizures (88–90). Treatment with the strong mAChR agonist pilocarpine results in N-methyl-D-aspartate (NMDA) receptor (NMDAR)-mediated excitotoxicity of hippocampal glutamatergic neurons (91). The degeneration of glutamatergic neurons coincides with severe epileptic seizures. Pilocarpine-induced epilepsy in rodents is, therefore, generally considered the most accurate animal model of human TLE (92). Patients with severe epilepsy symptoms known as Rasmussen encephalitis show a humoral immune response to metabotropic Glutamate receptor 3 (mGluR3) (93), whereas the affected brain parts are also infiltrated by CTLs (94, 95). The CTLs isolated from the inflamed brain parts of patients show a limited TCR repertoire, suggesting that these CTL’s are clonally derived and recognize a single or few self-antigens (19, 96). Agonists of the metabotropic group II receptors (mGluR2 and mGluR3) inhibit glutamate release in synapses of the glutamatergic hippocampal neurons of epilepsy patients and are potent anticonvulsants against motor and absence seizures (97), indicating that a shortage of metabotropic group II receptors may cause the generation of seizures in epilepsy patients. Immunization of rabbits with mGluR3 induces epileptic seizures (98). Immunization of rabbits with mGluR3 (but not mGluR1, 2, 5, or 6) results in the development of seizures and histopathological changes that mimic Rasmussen’s encephalitis (99). These immunological and pharmacological studies indicate that mGluR3 is the most likely pSAg involved in the destruction of hippocampal and cortical neurons in epilepsy patients.

#### Schizophrenia

Schizophrenia patients suffer from delusions, hallucinations, a distorted awareness, and disorganized thinking during psychotic episodes. Schizophrenia is associated with immune responses and lymphocyte infiltrations in the hippocampus and a reduced hippocampal gray matter size (100–106). It has been suggested that reduced striatal glutamate signaling increases the risk of sensory overload and of exaggerated responses in the monoaminergic system, consistent with schizophrenia symptoms. NMDAR antagonists, such as phencyclidine (PCP), induce schizophrenia symptoms, whereas NMDAR agonists are schizophrenia medicines. Mice mutants with reduced NMDAR levels develop symptoms resembling schizophrenia, which can be overcome by treatment with NMDAR agonists (107, 108). This suggests that NMDAR is a candidate pSAg involved in the striatal damage of schizophrenia patients (109).

Among the acute autoimmune encephalopathies anti-NMDAR encephalitis is the most prevalent. Patients generally show epileptic and/or schizophrenic symptoms indicating that hippocampal glutamatergic circuits are affected. It has remained unknown whether NMDAR is the pSAg or a secondary self-antigen of the disease. The encephalitis is treated by removal of the coinciding ovary teratoma that presents hippocampal pSAgs or by using anti-inflammatory drugs. The few remaining therapy-resistant anti-NMDAR encephalitis patients represent epilepsy and/or schizophrenia patients depending on the disease symptoms shown (110).

### Group 4: Histaminergic NPDs

#### Tic Disease

Patients with a tic disease including the Tourette syndrome show spontaneous short muscle contractions resulting in movements (motor tics) or vocals (vocal tics). Markers of inflammation are found in the basal ganglia and in the peripheral blood. Histaminergic neurons in the basal ganglia are targeted by the patient’s immune system (111, 112). Mice with a knockout mutation in the histidine decarboxylase gene that lack the capacity to produce histamine show tic-like stereotypes (113). These genetic and pharmacological studies suggest that the histamine 3 receptor is the pSAg of TD (114).

### Group 5: Serotonergic NPDs

#### Depression

Depression comprises bipolar or manic depression and unipolar or major depression. Patients usually undergo periods of mania and/or often severe depression. Depression is associated with decreased serotonergic activity in the prefrontal cortex, amygdale and/or hippocampus, inflammation markers in the brain and periphery, and a reduced size of the prefrontal cortex (115, 116). Imaging and post mortem studies in patients with severe depression revealed damage of specific parts in the prefrontal cortex (117–119) associated with leukocyte infiltrates from the periphery (120, 121). Depression is caused by a shortage of 5-hydroxytryptamine (5-HT) transporter (5-HTT) activity resulting in overstimulation of glutamatergic neurons in the prefrontal cortex upon stress (122). Indeed, 5-HTT knockout mice and rats show depression-related behavior with impaired neural plasticity (123, 124), suggesting that 5-HTT is the pSAg of depression. Selective serotonin reuptake inhibitors (SSRIs) are widely-prescribed to treat depression (125). The genetic studies using 5-HTT knockout animals, therefore, seem to be in conflict with the pharmacological studies using SSRIs. A possible explanation for this discrepancy could be that SSRIs serve as 5-HT receptor (5-HTR) agonists rather than 5-HTT antagonists (126).

#### Anxiety

Anxiety includes general anxiety, panic disease, and phobias. Anxiety coincides with decreased serotonergic activities in the raphe nuclei in the brain stem (127, 128). Studies using mouse strains with knockout or conditional knockout mutations in genes involved in serotonin or 5-hydroxytryptamine (5-HT) metabolism and strains overexpressing these genes, combined with pharmacological studies using SSRIs revealed that anxiety is caused by a shortage of 5-HT perception, resulting in defective glutamate signaling in the prefrontal cortex upon stress. These studies suggest that one of the serotonin receptors, probably receptor 2C (5-HTR2C) is the pSAg of anxiety (129).

## Novel Immunotherapies to Treat NPDs

In our aging population, degenerative diseases associated with aberrant immune responses, such as diabetes mellitus, arthritis, atherosclerotic cardiovascular disease, chronic obstructive pulmonary disease, and NPDs, have become highly prevalent (130–132). For only a few degenerative diseases more or less effective treatments have become available. To date, psychiatric diseases are treated with neurotransmitter antagonists/agonists that dampen undesired behaviors. A number of anti-inflammatory drugs, such as cyclooxygenase inhibitors, minocycline, omega-3 fatty acids, and neurosteroids, ameliorate the symptoms of psychiatric diseases, confirming the role of the patient’s immune system in the pathogenesis (133). For the neurodegenerative diseases, anti-inflammatory drugs are not sufficient to ameliorate or significantly delay disease progression. Only MS drugs that inhibit the migration of lymphocytes from the periphery to the CNS decrease the frequency of disease relapses.

Overall, all current treatments, if available, non-specifically suppress inflammation and only decrease the frequency of relapses and thus alleviate disease symptoms. Long-term use of non-specific immuno-suppressing medication coincides with often severe adverse side effects and enhances the risk of developing cancer or autoimmune processes in other tissues. There is, therefore, an urgent need for novel treatments of degenerative diseases in general and NPDs in particular that specifically inhibit tissue damage caused by activated cells of the adaptive immune system, leaving the general immune response unaffected. In principle, this should be feasible since autoimmune diseases are acquired diseases by individuals with fully functional immune systems.

Restoration of the immune tolerance to the pSAgs involved in the autoimmune tissue destruction, also named reverse vaccination, has been a longstanding goal in autoimmunity research. This has been attempted by administration of the self-antigens or peptide fragments derived thereof to patients (134–137). However, naked proteins or peptides are rapidly degraded in the body. Linkage of peptides to nanoparticles to improve their stability results in a transient effect with a very narrow spectrum of activity. The results of such protein/peptide-based tolerization approaches pursued so far did not meet the expectations (138).

An efficient way to instruct immune cells for suppressing an autoimmune response is to let them produce the self-antigens involved in the autoimmune tissue destruction by introducing the self-antigen-encoding genes into these cells, in a non-inflammatory or tolerogenic environment, such as the liver.

For the best-studied autoimmune disease animal models (DM1, MS and RA), it has been shown that viral vector-mediated expression of the pSAgs of the disease protects the treated animals from the autoimmune disease both in prophylactic and therapeutic settings (139–142). Viral vector-mediated tolerization, e.g., reverse viral vector vaccination, therefore, has an enormous potential for effectively treating degenerative diseases, including NPDs.

To date, reverse viral vector vaccines have not been tested in the clinic. The main reason for this is the immunogenicity or lack of *in vivo* efficacy of the currently most popular viral vectors, adeno-associated viral vectors derived from adeno-associated virus (AAV), and lentiviral vectors derived from the human immunodeficiency virus type 1 (143, 144). AAV’s immunogenicity in humans, and as a result clinical inefficacy, will remain the major challenge for the approval of new AAV-based interventions. Therefore, in order to develop efficient *in vivo* gene therapies and to efficiently restore immune tolerance in patients with a degenerative disease, a new viral gene delivery vector is needed. A new vector suitable for use in reverse viral vector vaccinations should combine the *in vivo* efficacy of AAV vectors in animals with being non-immunogenic in humans.

Only for the well-studied autoimmune diseases, the pSAgs are known and have been used in preclinical reverse viral vector vaccination studies (139–142). For some NPDs, such as MS, AD, ALS, ADHD and epilepsy candidate pSAgs have been identified (see The Candidate Primary Self-Antigens of NPDs), but for other important NPDs no clear candidate pSAgs involved in disease progression have been identified. All patients with an autoimmune disease have an adaptive immune response to the pSAg of the disease. Immunization of animals with a pSAg of an autoimmune disease results in the development of the characteristic disease symptoms (23, 28, 37, 64, 98, 99). Therefore, in order to verify whether a candidate self-antigen is the pSAg of the disease, immunological studies as described above are needed.

Once the vector immunogenicity and pSAg identification hurdles are taken, reverse viral vector vaccination has the potential to yield a whole new range of therapeutics and even prophylactics addressing today’s major diseases, which will dramatically change the medicine landscape.

This is particularly relevant for the highly mortal and severely debilitating NPDs. Effective reverse vaccines will halt neurodegeneration in patients with a neurodegenerative disease and will stop chronic or flaring inflammation in the CNS of psychiatric patients rendering them amenable to existing psychotherapy.

## Author Contributions

PH wrote the review. HK and BH equally contributed in editing the manuscript.

## Conflict of Interest Statement

The authors declare that the research was conducted in the absence of any commercial or financial relationships that could be construed as a potential conflict of interest.
